# GLOSSary: the GLobal Ocean 16S subunit web accessible resource

**DOI:** 10.1186/s12859-018-2423-8

**Published:** 2018-11-30

**Authors:** M. Tangherlini, M. Miralto, C. Colantuono, M. Sangiovanni, A. Dell’ Anno, C. Corinaldesi, R. Danovaro, M. L. Chiusano

**Affiliations:** 1 Stazione Zoologica “Anton Dohrn”, Villa Comunale, 80121 Naples, Italy; 2 Dipartimento di Scienze della Vita e dell’Ambiente, Polytechnic University of Marche, Via Brecce Bianche, 60131 Ancona, Italy; 3 Dipartimento di Scienze e Ingegneria della Materia, dell’Ambiente ed Urbanistica, Polytechnic University of Marche, Via Brecce Bianche, 60131 Ancona, Italy; 4 Dipartimento di Agraria, University of Naples “Federico II”, via Università 100, 80055 Portici, Italy

**Keywords:** Tara Ocean project, Marine metagenomics, 16S rRNA, Taxonomic analysis

## Abstract

**Background:**

Environmental metagenomics is a challenging approach that is exponentially spreading in the scientific community to investigate taxonomic diversity and possible functions of the biological components. The massive amount of sequence data produced, often endowed with rich environmental metadata, needs suitable computational tools to fully explore the embedded information. Bioinformatics plays a key role in providing methodologies to manage, process and mine molecular data, integrated with environmental metagenomics collections. One such relevant example is represented by the Tara Ocean Project.

**Results:**

We considered the Tara 16S miTAGs released by the consortium, representing raw sequences from a shotgun metagenomics approach with similarities to 16S rRNA genes. We generated assembled 16S rDNA sequences, which were classified according to their lengths, the possible presence of chimeric reads, the putative taxonomic affiliation. The dataset was included in GLOSSary (the GLobal Ocean 16S Subunit web accessible resource), a bioinformatics platform to organize environmental metagenomics data. The aims of this work were: i) to present alternative computational approaches to manage challenging metagenomics data; ii) to set up user friendly web-based platforms to allow the integration of environmental metagenomics sequences and of the associated metadata; iii) to implement an appropriate bioinformatics platform supporting the analysis of 16S rDNA sequences exploiting reference datasets, such as the SILVA database. We organized the data in a next-generation NoSQL “schema-less” database, allowing flexible organization of large amounts of data and supporting native geospatial queries. A web interface was developed to permit an interactive exploration and a visual geographical localization of the data, either raw miTAG reads or 16S contigs, from our processing pipeline. Information on unassembled sequences is also available. The taxonomic affiliations of contigs and miTAGs, and the spatial distribution of the sampling sites and their associated sequence libraries, as they are contained in the Tara metadata, can be explored by a query interface, which allows both textual and visual investigations. In addition, all the sequence data were made available for a dedicated BLAST-based web application alongside the SILVA collection.

**Conclusions:**

GLOSSary provides an expandable bioinformatics environment, able to support the scientific community in current and forthcoming environmental metagenomics analyses.

**Electronic supplementary material:**

The online version of this article (10.1186/s12859-018-2423-8) contains supplementary material, which is available to authorized users.

## Background

Environmental metagenomics is a challenging approach that has rapidly expanded in the last decade thanks to advanced high throughput sequencing technologies. Massive amounts of sequence data are being produced, accompanied by information annotating spatial localization of the sampling sites, as well as other useful data related to the environmental conditions (e.g. nutrient concentrations, temperature [1]), which represent some of the driving factors that can shape community structures by resource partitioning [2] and influence biological processes [3].

Metagenomic approaches are mainly focused on the analysis of microbial communities, fostering a deeper characterization of prokaryotic and eukaryotic diversity. The exploration of marine microbial communities is expanding to integrate classical phylogenetic analyses with information from environmental data and spatial-temporal variation of such communities [4]. Important efforts, indeed, aims at considering multilevel aspects, such as molecular, biological, physical, and chemical data for holistic approaches to the study and the understanding of the processes which regulate Earth’s biogeochemical cycles and climate [5]. The global ocean exploration that started in the last two decades is contributing enormously to this aim. The Sorcerer II expeditions (2003–2010) and the Malaspina expedition (2010–2011) carried out surveys for exploring microbial diversity from the ocean surface down to bathyal depths (> 1,000 m). The Tara Oceans Expedition (2009–2013) is by far the most recent and largest expedition, aimed at investigating microbial and eukaryotic diversity at global scale [5]. These research efforts result in the production of a huge amount of data; as an example, the Tara Oceans expedition resulted in the collection of over 35,000 samples of seawater and plankton, with most of the data represented by sequence data [6]. The analysis of such “Big Data” collections requires huge collective efforts, addressed by several and independent research teams [6–11]. However, the amount of produced sequence data requires dedicated infrastructures to be fully exploited and properly analysed, and this represents one of the most daunting challenges in the field of environmental ecology. Organising such collections and their precious and multifaceted information content by suitable bioinformatic tools represents a key step to foster both data mining and collaborative research and discovery [5, 6], with relevant impact for a better understanding of microbial diversity and factors influencing its distribution.

Previous attempts at the creation of sequence storage and analytical platforms, dedicated to the study of environmental sequence data, included the CAMERA database [12], which is now discontinued; the VAMPS system [13], for ribosomal sequence analysis; the MG-RAST server for metagenomics and metatranscriptomics [14]; and QIITA [15], which acts as a sequence analysis platform serving as a repository for the Earth Microbiome Project [16]. However, none of them currently supports metadata-enabled dataset exploration.

To test a novel computational framework for the management and the analysis of environmental omics collections, we designed a document-oriented schema-less database together with a web interface with the aim of setting up: i) a suite using alternative computational approaches to manage challenging metagenomics data; ii) a user-friendly web-based platform to allow the integration of environmental metagenomics sequences and of the associated metadata; iii) an appropriate bioinformatics platform to support the analysis of 16S rDNA sequences while exploiting reference datasets, such as the SILVA data collection [17].

Although public sequence collections have been recently distributed through the European Nucleotide Archive [18] by the Tara Ocean consortium, 16S sequence data are only available in the form of raw reads, also providing a basic summary of the associated diversity. However, to our knowledge, no sequence curation is provided yet. Therefore, we decided to start the implementation of the platform here proposed including data from this relevant collection. We considered the 16S miTAGs [8, 19] originally retrieved from shotgun sequencing data by means of specialized HMM-based pipelines focused on ribosomal 16S rRNA gene sequences within the 0.22–1.6 μm / 0.22–3 μm size fractions of water samples processed by the Tara Ocean consortium. We processed the miTAGs data, and classified complete or partial sequences, as well as potential chimeric and chimera-free sequences, providing taxonomic affiliation by means of the SILVA 16S gene sequence database. We organized all the sequence data and their associated metadata in the platform and designed suitable analytical tools to browse the entire collection, aiming to support in-depth exploitation of this precious resource.

## Implementation

### Data availability

Tara miTAGs libraries, representing 16S rDNA sequence tags extracted from Tara raw reads (thus not including 18S sequence data), as well as all the metadata related to all libraries and to the specific Tara stations, were downloaded from the Tara Ocean companion website (http://ocean-microbiome.embl.de/companion.html). MiTAG libraries were produced from shotgun sequence data from the 0.22–1.6 μm / 0.22–3 μm size fractions of water samples generated by the Tara Ocean consortium, by means of specialized HMM-based pipelines focused on prokaryotic ribosomal 16S genes. Sampling depths are reported per library and correspond to surface oceanic waters (“SRF”), epipelagic waters (“MIX”), waters sampled at the deep-chlorophyll maximum (“DCM”) and mesopelagic waters (“MES”) [6, 7]; libraries were assigned to stations, depth layers, marine biomes and oceanic provinces according to the metadata provided in [6].

### Assembly and annotation pipeline

MiTAG sequences from each library were independently assembled by the MEGAHIT software [20]. MEGAHIT was chosen because it achieves a reasonable resolution of 16S rRNA genes micro-diversity (intended as 16S gene sequence variants with an overall similarity higher than 97%, e.g. 1–3 nucleotides) during the assembly, as reported in [21]. MEGAHIT was used with the following settings: k-mer lengths from 21 to 99 (with a k-step of 10) and a minimum coverage of 2 sequences (to maximize assembly sensitivity). Other parameters were left as default. This allowed us to discern prokaryotic strains potentially differing for few nucleotides in their 16S genes, thus broadening our view of prokaryotic diversity in marine habitats.

To detect sequences presumably used to build each assembly, and remove sequences with ambiguous mapping, miTAG sequences from each library were indipendently mapped to all assembled contigs using the BBMap tool (https://sourceforge.net/projects/bbmap/), with a minimum identity threshold of 97% (minid = 97) and all the other in the default settings. Mapped and unmapped (i.e. singletons) sequences were therefore associated to each library from each Tara station and depth. The percentage of mapped miTAGs, i.e. the number of miTAGs mapped to each contig versus the total library size, was used as a proxy for defining the relative abundance of a contig in a library. This also represents the *coverage* of the contig, and is reported in the header associated to each contig sequence when exploring the database. Visualization of the resulting data and statistical analyses were performed within the R environment [22].

To trim the assembled contigs and remove non-16S portions, the *ssu_finder* function of the CheckM package [23] was used. From this dataset, all sequences with a minimum length of 900 bp were kept using the PRINSEQ tool [24] and were defined as “*long*” sequences; sequences with a length ranging from 800 to 899 bp were also recovered by the same tool and classified as “*medium*”. Sequences shorter than 800 nucleotides, that confirmed their similarity to 16S RNAs, were defined as “*short*.”

*Long* and *medium* contigs were annotated using the *assign_taxonomy* function of the QIIME package (v1.9; [25]) using the SILVA v128 [17] database as a reference. Both sets of contigs were independently checked for possible chimeras using the VSEARCH package [26], again using the SILVA v128 database as a reference. We therefore classified all *long* and *medium* contigs as chimeric (“*chimera*”), non-chimeric (“*chimera free*”) and “*borderline*”, accordingly.

**Fig. 1:**
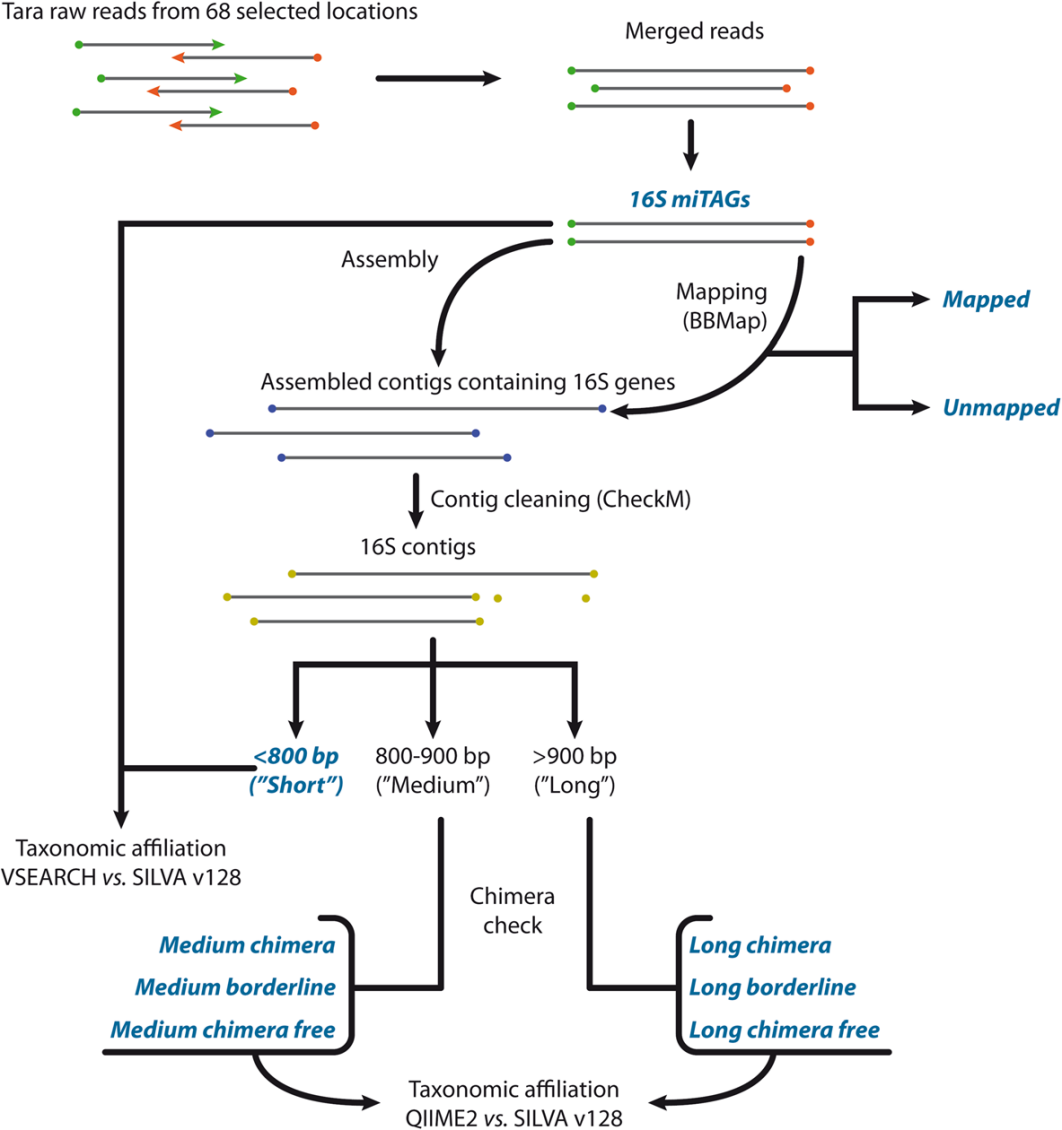
Schematic representation of the pipeline implemented to process and annotate the data included in the GLOSSary database. Data included in the database and their classification are indicated in blue

Taxonomy affiliations for short contigs (< 800 bp), as well as for the miTAG sequences, were obtained by a similarity search versus the SILVA database using the VSEARCH package using an identity cutoff of 80% and keeping the best hits. The complete pipeline is shown in Fig. 1.

### Database organization and query system

**Fig. 2:**
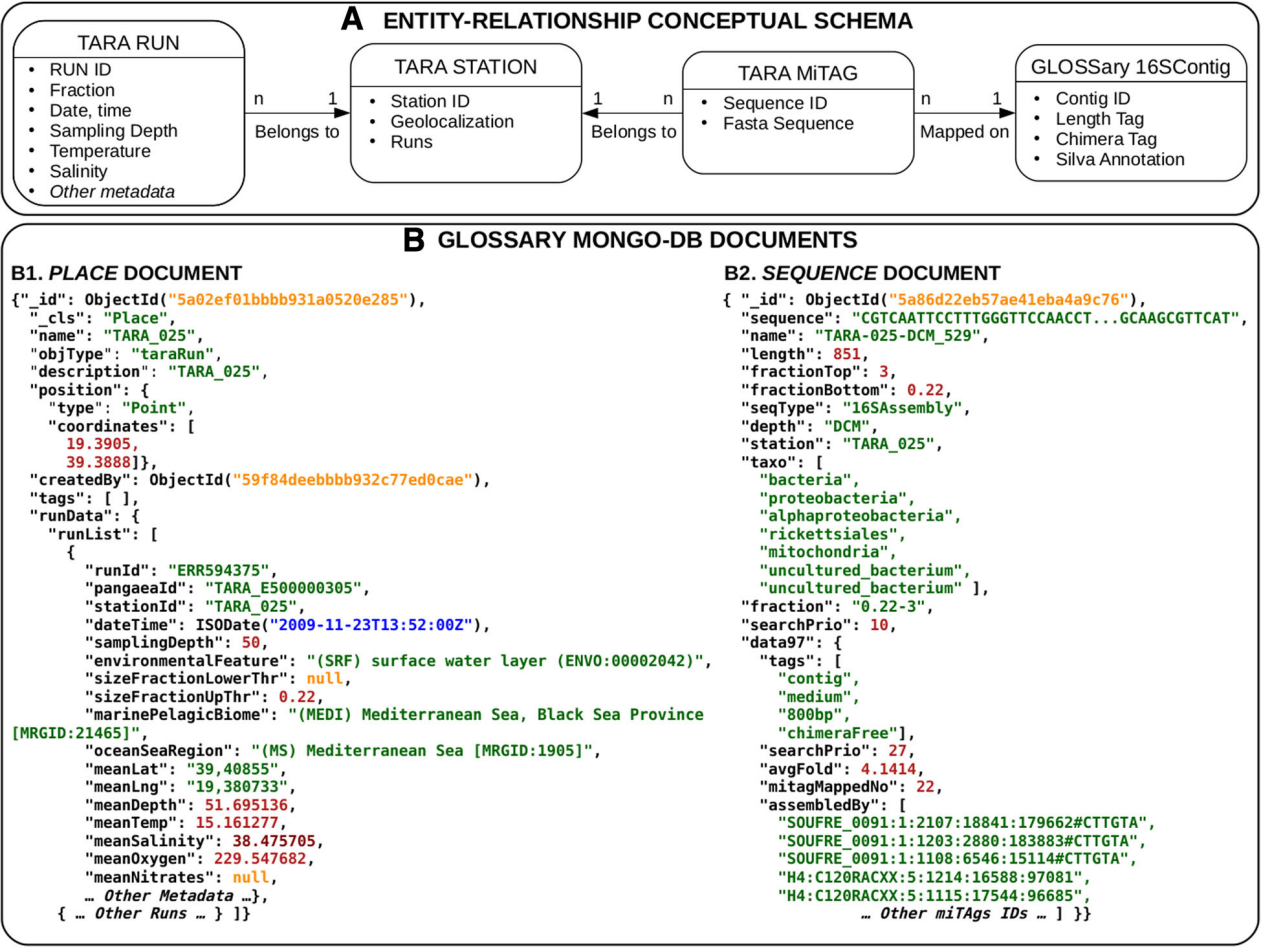
Conceptual and implemented architecture of the *GLOSSary* database. **a** Entity-Relationship diagram of the *GLOSSary* data. The Tara sequences and metadata are organised in runs (*TARA RUN*), each belonging to a specific station (*TARA STATION*). *TARA MiTAG*s are short sequences obtained by merging the paired end reads matching on the Silva 16S database, grouped by station, depth and fraction. The *GLOSSary 16SContigs* are longer sequences obtained by re-assemblying the MiTAGs with the glossary pipeline, and mapping them to the Silva database. **b** The *GLOSSary* Mongo-db document organization. The data presented in panel A have been denormalised and reorganised in two main documents: PLACE (*B1*) and SEQUENCE (*B2*). The PLACE document holds all the information on the Tara stations and associated runs and metadata. The SEQUENCE document holds the *GLOSSary* 16S contigs and the unassamblied MiTAGS nucleotidic sequences, together with the associated metadata and, for the 16SContigs, the taxonomical information and assembled MiTAGS identifiers

The database was implemented using MongoDB [27], a document-oriented NoSQL Database management system. Indeed, MongoDB permits to efficiently store and retrieve semantically similar data having highly different structures. More in detail, the database underlying GLOSSary holds together miTAG and contig sequences (i.e. semantically similar objects), although each type could have a largely different metadata set (i.e. structure). Data were de-normalized and split over several collections, taking into account query performances as one of the main criteria. Starting from a pre-defined set of queries and data-browsing requirements, data collections were built together with a first set of indexes to allow fast data retrieval. The sequences were stored in a dedicated collection. Each sequence data object was augmented with a set of metadata, which differ across miTAG or contig sequences. The sequences were then tagged using an indexed array of keywords, representing query filtering criteria exploited in the search engine. It is worth noting that each sequence object can be used to store large amount of data (up to 16 MB, as for the default per-document object limit in MongoDB). Due to the unstructured nature of the available data, a mix of single field indexes, geospatial indexes and text indexes were defined for each data collection to optimize data querying. To speed-up searches on large set of sequences (in the order of Millions of objects) a query caching approach was developed. The database general schema is represented in Fig. 2. The database can be accessed by a suitable query system through a web interface realized with a Pyramid stack (https://trypyramid.com/), a Python-based web framework. The web frontend was developed using HTML5, Bootstrap (https://getbootstrap.com/) and Jquery (https://jquery.com/).

### BLAST service

A BLAST service was implemented to allow similarity searches on raw 16S miTAG sequences, the contigs and the 16S full-length gene sequences from the SILVA database (v128) [17]. The service was developed using the Sequence Server software [28].The BLAST service allows the user to compare one or more query sequences (provided in FASTA format) against different partitions of the data collections included in the platform. The resulting hits are cross-linked to the web platform above described.

## Results

### Data content

The total amount of sequencing data associated with the 139 miTAG libraries available on the Tara Ocean portal corresponded to 14,129,971 miTAG sequences. Of these, 3,489,675 reads (representing, on average, ca. 25% of the raw miTAGs in each library) were assembled in contigs, according to the in-house implemented pipeline (Fig. 1). On average, ca. 5% of the total resulting contigs (103,954 sequences) was greater or equal to 800 bp in length and identified as potentially non-chimeric. An additional table shows this in more detail (Additional file 1).

The number of miTAG libraries obtained for each marine province of the Tara sampling was very variable, with the highest number of libraries (27) in the South Pacific Subtropical Gyre Province (SPSG). Surface layers (“SRF”) were represented across all provinces, but deeper layers (i.e. deep-chlorophyll maximum (“DCM”), epipelagic mixed waters (“MIX”) and mesopelagic waters (“MES”)) were not: indeed, a total of 63 SRF, 42 DCM and 30 MES libraries were produced, and some layers were entirely missing (e.g. the DCM layer from the Gulf Stream province) A visualization of this unevenness is shown in an additional figure (Additional file 2).

The number of sequences included in each miTAG library was highly variable (Additional file 1), ranging from the small content of 39410 sequences in the TARA-066-DCM library (Benguela province, DCM layer) to the 186,898 sequences in the TARA-064-SRF library (Eastern Africa Coastal province, SRF layer). However, more than 24% of the sequences across all libraries (up to 43%) were successfully assembled.

### Platform organization and accessibility

The platform is organized in two independent partitions: 1) a database including sequence data and metadata, and 2) a BLAST based platform including miTAGs and contigs, together with the 16S full-length sequences from the SILVA database (v128).

All raw and assembled sequences (i.e. miTAGs and contigs) were uploaded into a MongoDB Database management system (DBMS). The sequences are characterized by a “tag”, i.e. a descriptive string reporting the classification of several features such as: i) mapped and unmapped status for each miTAG; ii) contig classification (*long*, *medium* and *short*) and iii) contig contamination level (*chimera free*, *chimera* and *borderline*). Information about the metadata (such as Tara station ID, depth and fraction) associated to each miTAG were kept and used as added “tags”.

**Fig. 3:**
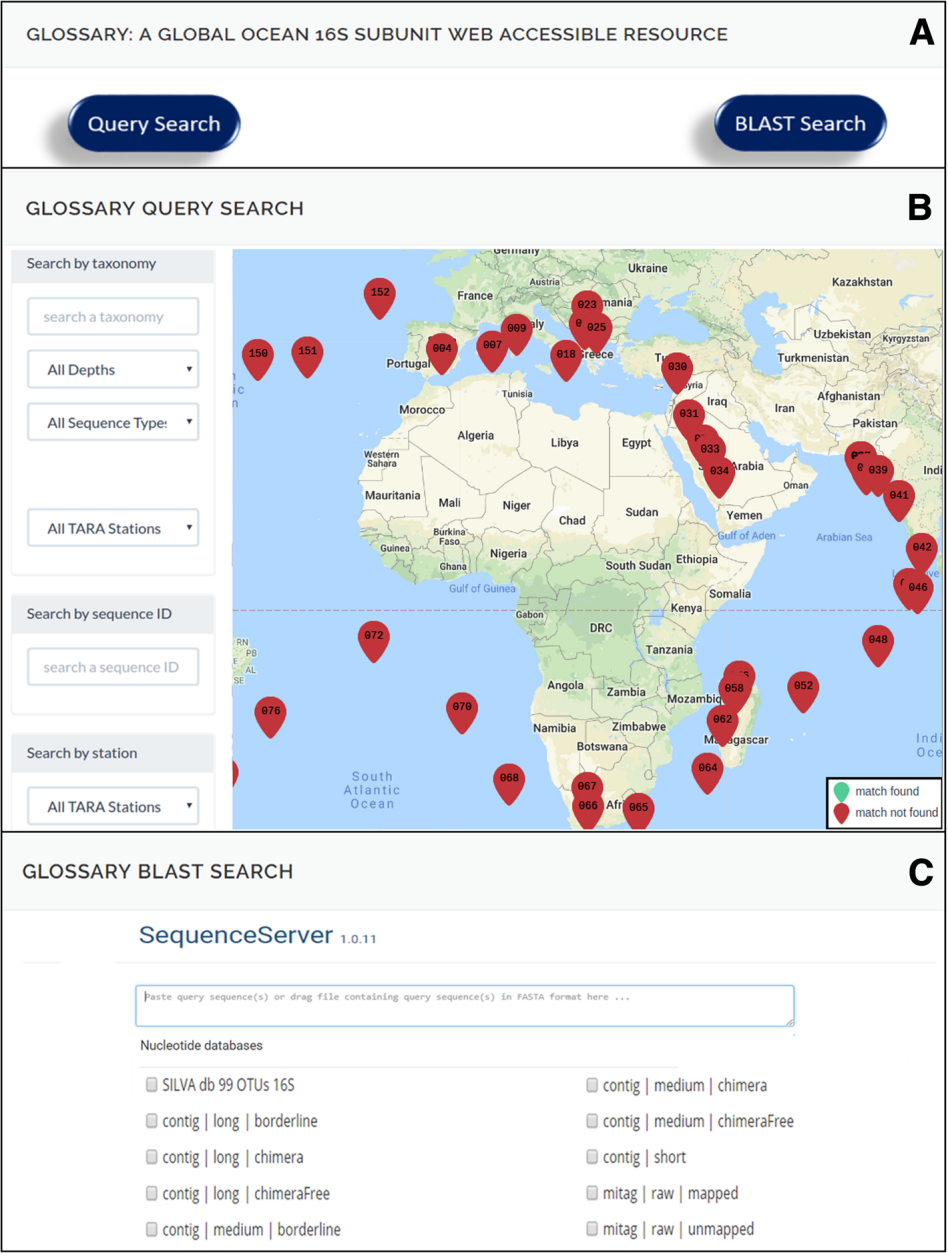
The GLOSSary web interface. **a** Links to the “Query Search” and the “BLAST Search” are accessible by the dark blue boxes in the main page of the GLOSSary platform. **b** The “Query Search” main page: “Search by taxonomy”, “Search by sequence ID” and “Search by station” are shown. Red pointers on the interactive geographic map indicate the Tara stations by number. Each pointer is linked to the station information details. **c** The “BLAST search” main page. The sequence server interface permits to perform BLAST searches on one or multiple data subsets (Tags) of the GLOSSary database. The SILVA database is also querable in this partition

Both the miTAGs and the 16S contigs can be explored through the web page http://bioinfo.szn.it/glossary (Fig. 3a). Two main sections are accessible: a “Query Search” page (Fig. 3b) and a “BLAST Search” service (Fig. 3c).

The “BLAST Search” service allows the user to compare one or more query sequences (to be provided in FASTA format) against different subsets of the data included in the GLOSSARY database. Moreover, among the datasets made available in the BLAST partition, we also included the SILVA 16S full-length sequences, to allow cross comparisons between the entire data collection organized in the database and a reference database (Fig. 3c). The user can select one or more of the included collections, and the alignments in the classical BLAST output format are shown.

In the “Query Search” area of the platform, three main entry points are currently available. The “*Search by taxonomy*” allows a query by keyword based on taxonomical classification; the “*Search by sequence ID*” allows to use miTAGs or contigs identifiers to access each single sequence features; the “*Search by station*” allows to access to all the runs and the miTAGs belonging each Tara station.

An interactive geographic map can be also exploited independently, or can accompany each query by the appropriate result. The map changes according to the results of each query, highlighting in green all stations containing sequences associated with the searched keyword, thus permitting an immediate and dynamic view of the geographical distribution of the stations associated to each query.

**Fig. 4:**
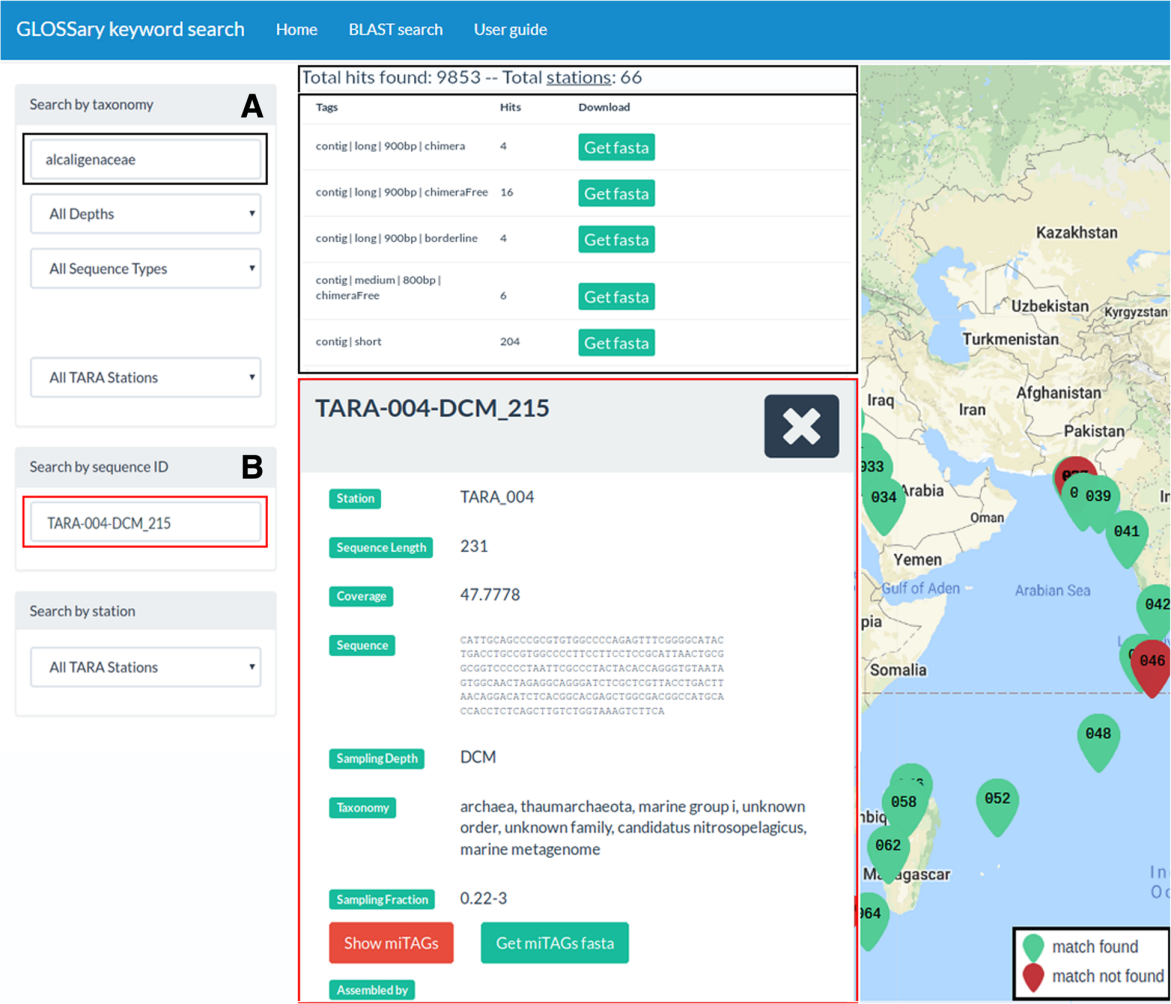
Details on example queries. **a** Selecting in the “Search by taxonomy” section the “*Alcaligenaceae*”, “*All Depths*”, “*All Sequence Types*” and “*All TARA Stations*” options, the total number of elements found for each Tag is shown. A button allows to download the sequences belonging to each Tag (“*Get fasta*” green boxes). The Tara stations associated to each query are also shown on the map by green pointers. **b** Typing in the “Search by sequence ID” field the “*TARA-004-DCM_215*” contig ID, several details are shown including the sequence, its length, the taxonomic affiliation, the Tara station it belongs to, the sampling depth, the sampling fraction. It is also possible to view the list of all the miTAGs assembled on that contig (“*Show miTAGs*” red box), or to download the corresponding miTAGs FASTA file (“*Get miTAGs fasta*” green box)

In detail, the “*Search by taxonomy*” query field allows to perform the search specifying the available taxonomic affiliations while typing one or more chars. A dropdown list appears accordingly supporting the selection. The list includes all the taxa that are associated to at least one of the sequences included in the database (Fig. 4a). Furthermore, it is possible to further specify the query by selecting: i) the sampling depth: “*Surface Oceanic*” (“SRF”), “*Deep-Chlorophyll Maximum*” (“DCM”), “*Epipelagic*” (“MIX”) and “*Mesopelagic*” (“MES”); ii) the sequence type, i.e. “*All Sequence Types*”, “*miTAGs*” or “*16S Contigs*”; iii) when searching for “*16S Contigs*” it is possible furthermore to select the contigs by length (“*All Contigs Length*”, “*Long (> 900bp)*”, “*Medium (> 800bp and < 900bp)*”, “*Short (< 800 bp)*”); iv) when searching for “*miTAGs*” it is possible to choose among “*All miTAGs*”, “*Mapped*” or “*Unmapped*” sequences, thus identifying all sequences types including those that resulted as singletons at the given similarity threshold; v) the Tara station ID: “*All TARA Stations*” or, selecting from the dropdown list, a specific station ID.

The query results are visualized next to the search boxes and provide information on the total number of hits and stations found for the selected criteria (topmost line, Fig. 4a) and a table, showing the number of hits found for each tag. Result sequences are downloadable in a FASTA formatted file (Fig. 4a), whose headers contain information on the Tara station from which the sequence was reconstructed, alongside with size fraction, depth layer, taxonomy, sequence tags and coverage information. By clicking with the mouse on the “total stations” link in the summary line, it is possible to visualize the list of hit counts for each station.

By searching for a specific sequence ID in the “*Search by sequence ID*” box it is possible to obtain information on the sequence and its length, the corresponding Tara station, the sampling depth and fraction, and the complete taxonomic affiliation. In the case of contigs the coverage is available, and the miTAGs sequences mapped at the specific threshold are also accessible, whereas, in the case of miTAGs, their status in terms of mapped or unmapped to contigs is reported too (Fig. 4b).

**Fig. 5:**
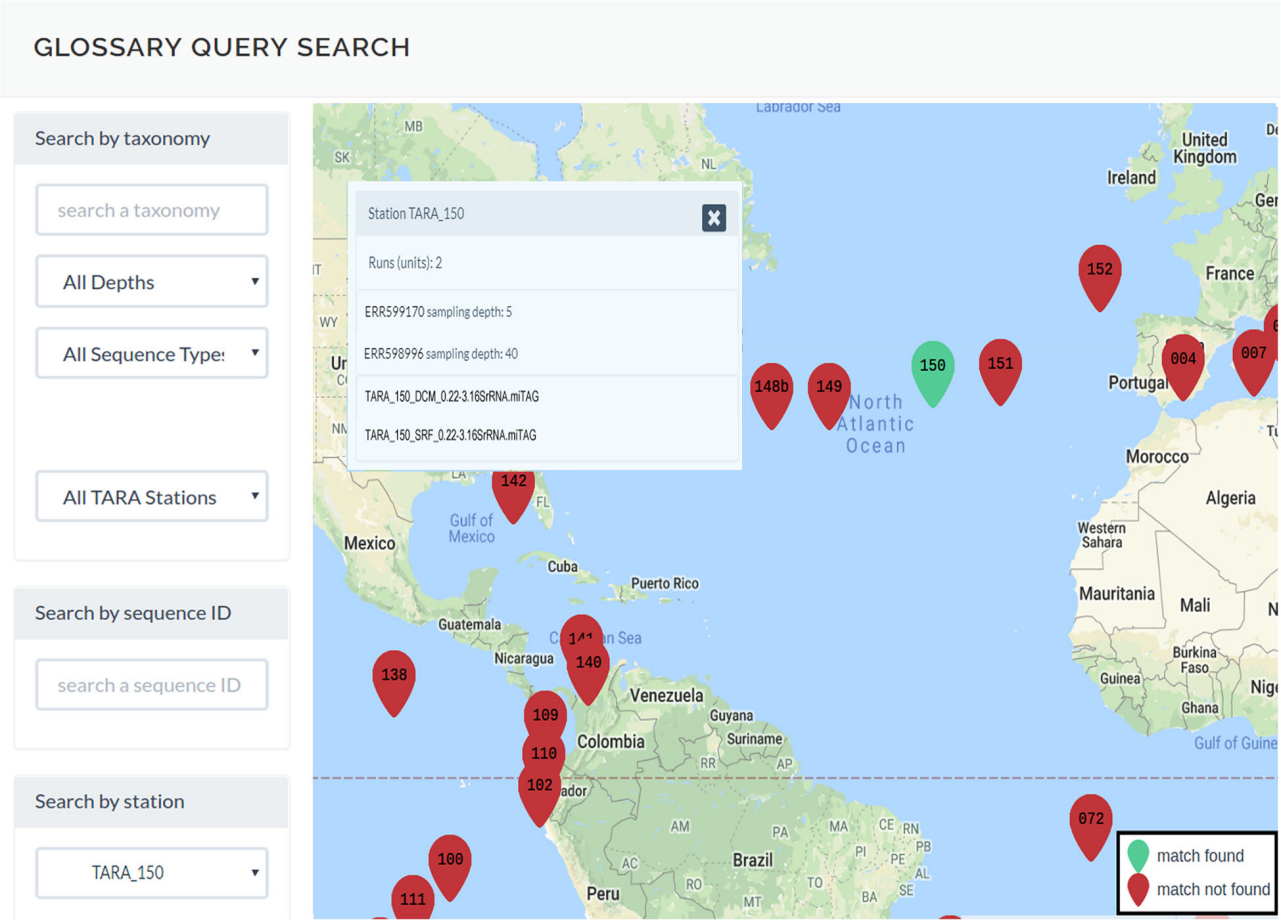
Example of a search by station. Selecting in the “Search by station” section a Tara station ID (such as “*TARA_150*”, green map pointer), the list of available runs and miTAGs archives (one for each depth) for that station is shown. By clicking on the run name the user will be redirected to the download page of the NCBI SRA section, whereas by clicking on the miTAGs file name it is possible to directly download the FASTA file of the miTAGs sequences for that station and depth

Finally, searching by Tara station IDs in the dropdown menu of the “*Search by station*” box allows to obtain the list of the raw runs for each station, each linked to the NCBI SRA download page, as well as the list of the miTAGs sequences (as FASTA archives) organised by depth (Fig. 5).

For all the search types, the resulting stations are highlighted in green on the map. The map itself can be interactively explored: holding the left button of the mouse it is possible to grab and move the map, and by clicking on a station the related information will be shown.

### Data analysis

The preliminary overview of our results reveals that the percentage of high-quality 16S fragments assembled (all sequences > 900 bp, and chimera-free vs. the total number of contigs assembled) was rather homogeneous across different water depths, ranging from an average of 2.6% for all MES layers to an average of 4.4% for all SRF layers (Additional file 1). Interestingly, a high fraction of high-quality fragments (> 6%) was assembled from both the Antarctic province and the Indian Monsoon Gyres province (from SRF and MES layers, respectively), whereas the lowest fraction of high-quality gene fragments was assembled from the MES layer of the Chile-Peru Current Coastal province. No significant relationship was found between the percentage of assembled sequences and the number of high-quality contigs identified.

**Fig. 6:**
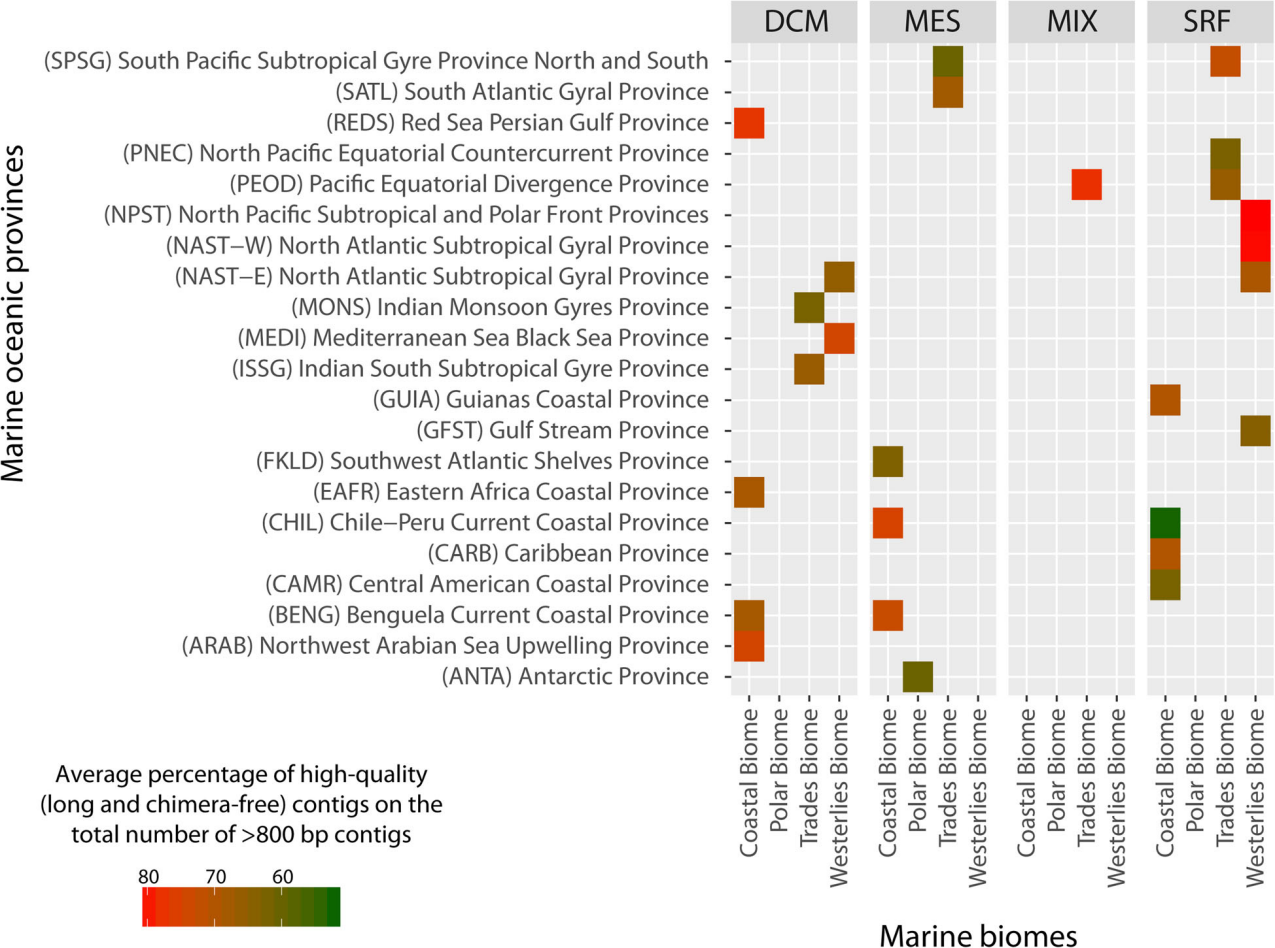
Percentage of long (> 900 bp) chimera-free contigs on the total number of assembled contigs from each marine oceanic province, aggregated per sampling depth (surface: SRF; epipelagic: MIX; Deep-Chlorophyll Maximum: DCM; mesopelagic: MES) and marine biomes (costal, polar, trades and westerlies)

The number of putatively chimeric contigs represents a variable fraction of the total number of *long* contigs, ranging from 0% up to 32% for contigs longer than 900 bp to more than 60% of the contigs between 800 and 900 bp, across all samples (Fig. 6).

**Fig. 7:**
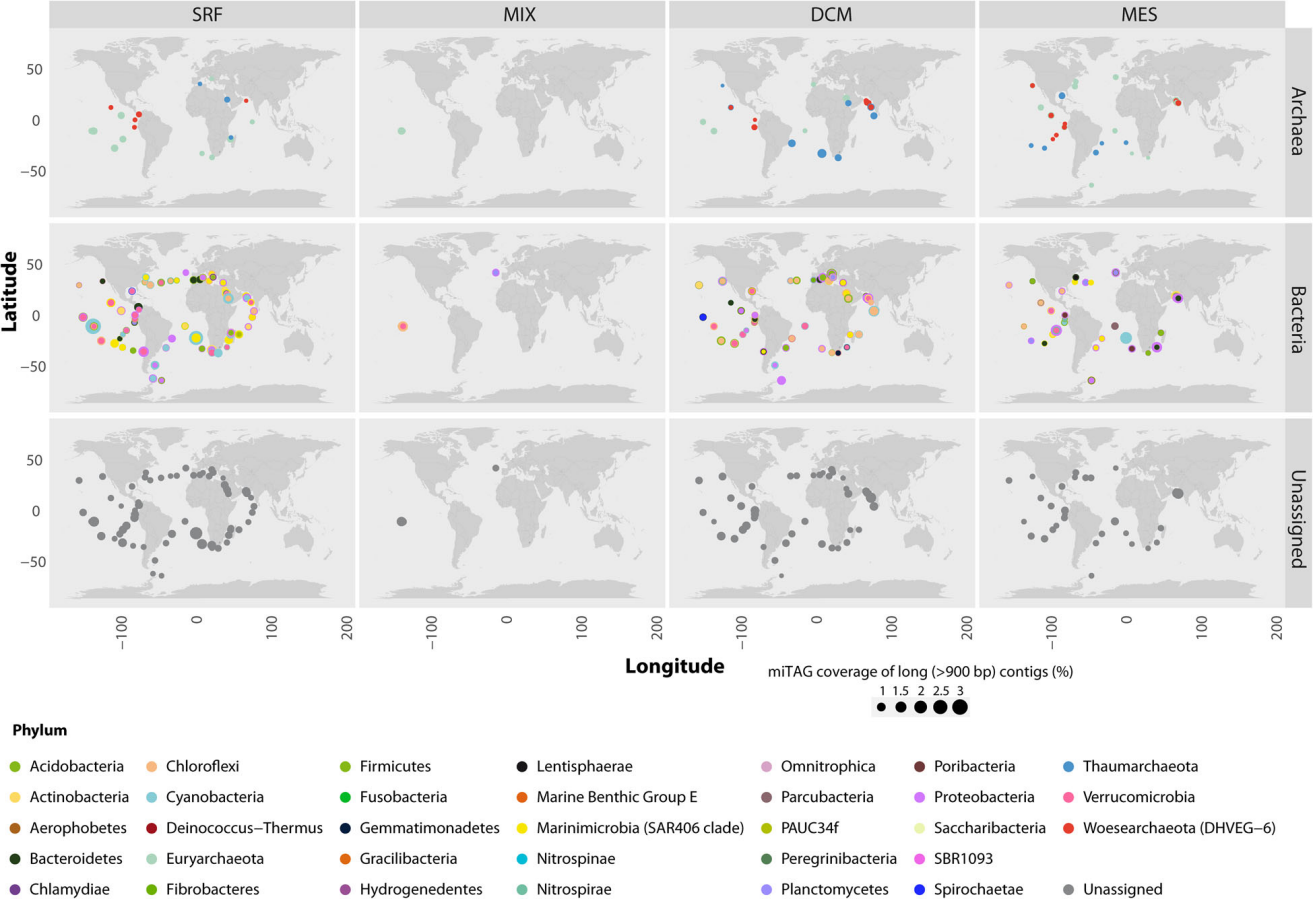
Geographical distribution and coverage of long (> 900 bp) chimera-free contigs affiliated with major prokaryotic lineages. Colour identifies the prokaryotic affiliation, and size represents the coverage. Results are classified in Archaea, Bacteria and Unassigned and grouped for sampling depth (surface: SRF; epipelagic: MIX; Deep-Chlorophyll Maximum: DCM; mesopelagic: MES). Points are placed on the maps based on the Tara station position

We analysed the geographical distribution of *long* contigs and represented their relative abundance in terms of number of miTAG per contigs per phylum. Contigs which could not be assigned to any known phylum were also reported as “Unassigned” (Fig. 7). Overall, the average coverage of *long* contigs reproduced known spatial patterns of prokaryotic abundance in the global ocean where Archaea show a limited distribution in comparison with Bacteria. Interestingly, “Unassigned” contigs are rather frequent and represented in the overall collection, revealing the wideness of still uncovered information on possible prokaryotic phyla.

## Discussion

Previous efforts carried out in the analysis of environmental datasets, such as the Earth Microbiome Project [16, 29] and the Global Ocean Sampling [30], produced an astounding amount of sequence data from several environmental samples across the world. Global scale studies can produce an overwhelming amount of data, based on consistent methodological approach, which for the Tara Ocean approach relied also on the use of shotgun genome sequencing strategies. This, in general, allows to collect an important amount of genomic data along with precious informative content [7], thus expanding the targeted sequencing approach previously employed in the Global Ocean Sampling cruises [30]. However, the evaluation of taxonomic diversity across huge datasets with different sampling sizes [19] requires well-calibrated, robust and appropriate methods to get reasonable results. The selection of appropriate sequencing approaches is driven by compromises in terms of sequencing costs and scientific outputs: the possibility of getting a wider information content with “shotgun” metagenomics than with targeted sequencing may produce novel insights on distribution of species and on their roles in the ecosystems, although the definition of OTUs may be not accurate and capturing the real diversity of prokaryotic taxa may be hard [29, 31].

Rich metadata collections, provided alongside with molecular data associated with environmental ‘omic studies, add a further layer of complexity to the analysis. Since the number of similar initiatives is widening (e.g. the Earth Microbiome Project [16, 29], the MicroB3 network [32], the Tara Ocean project [5–7], the Ocean Sampling Day initiative [33], we focused on possible computational approaches to appropriately exploit the information content from shotgun sequencing even in relation with environmental sampling metadata.

To explore the possible computational frameworks that could support appropriate organization and access to these data types, integrating sequence data information and environmental metadata, we designed a dedicated platform, based on a document-oriented NoSQL database management system (DBMS) [27].

We focused on the management and maintenance of information on 16S rDNA sequences considering also the possibility to organize collections of heterogeneous quality, since these data type may occur more frequently in shotgun metagenomics and, nevertheless, they represent precious information content to be investigated too.

The starting setup was implemented considering the Tara Ocean dataset, since these data were recently released [6] and they still need a deeper and wider exploration, also exploiting the wide environmental dataset contextually collected. We reconstructed putative 16S rDNA contigs by assembling miTAGs, representing 16S sequence tags identified and extracted by the Tara Ocean Consortium. The MEGAHIT assembler was chosen to this aim because of its ability to solve 16S micro-diversity, thus potentially allowing to discriminate among prokaryotic strains, differing for identities < 97% in their 16S rDNA sequence [21]. Moreover, the employed strategy allowed maintaining abundance information: in fact, the global abundance of major prokaryotic taxa was similar to that highlighted in previous works [33, 34]. Indeed, although the number of long contigs was < 10% for each library here considered, yet we managed to produce more than 4000 long, chimera-free contigs, which can be used for more complex queries and might allow the exploration of new hypotheses in microbial ecology [7, 35–39].

The presence of putatively chimeric sequences could be due either to miss-assemblies (typically caused by the combined effect of the different degree of variability within the prokaryotic 16S locus and the relatively short length of the raw sequences, which can lead to assembly of non-related sequences) or to genuine gene novelty (thus representing 16S prokaryotic ribosomal genes with still unclear phylogenetic placement). Although this issue is beyond the scope of the present work, it might be worth of future endeavours to fully characterize the real diversity underlying such datasets. New research direction involving phylogenetic diversity analyses on the present dataset might include a more thorough investigation on the taxonomic assignment of *short* contigs and raw reads, taking into account the composition of conserved vs. hypervariable regions of prokaryotic 16S genes, as well as the presence and characterization of mitochondrial and plastid sequences (which are currently marked as affiliating to either mitochondrial or chloroplast gene sequences according to the SILVA data description).

The BLAST server was implemented using the Sequence Server software [28]. Indeed, this approach allows us to set-up an in-house BLAST service, accessible from the web and with a standard BLAST NCBI-like input and output formats, permitting user driven selections of different data collections. As an example, the possibility to explore in parallel results from the SILVA database [17] and from the in-house implemented collections permits to cross-check sequence relationships as well as to access sequences information along with environmental metadata.

The choice of MongoDB as an alternative approach to more conventional relational DBMS technologies is an emerging trend in bioinformatics, as demonstrated by other similar research projects [40]. This is due to different reasons: i) the huge size of the dataset, demanding replication and data sharing to guarantee safety and performances, as well as scalability; ii) the inhomogeneity of the data collections, which can be much more easily addressed using a schema-less DBMS; iii) the possibility to query geospatial data natively; iv) the possibility to quickly re-organize data collections, allowing for database updates and changes. Since the MongoDB structure and capabilities meet the aforementioned requirements, it has been chosen as the document database for this study.

Similar platforms as the one here proposed are not new to science: complex systems, such as MG-RAST [14], allow researchers to upload, store, analyse and compare metagenomics samples on a global scale. Ribosomal sequence repositories such as the SILVA database [17] also allow scientists to make queries using a proprietary alignment software to identify similar sequences (for a sequence query-based search) or to simply browse the database to download sequences of interest. However, although such web-based systems have become part of standard practices in both shotgun and targeted metagenomics efforts, none of them allows to interactively exploit environmental metadata, which is of paramount importance for ecological studies. Indeed, for instance, MG-RAST allows users to search for specific samples or projects by means of MIMARKS-based metadata [14, 41], which are provided during the submission by data providers, although the environmental metadata are not accessible by straightforward queries on sequence data. The SILVA database [17] provides users with details on sequence data processing and production, but does not store any contextual data in the sequence dataset. The MarRef database [42], on the contrary, provides a rich set of metadata concerning both prokaryotic species features and environmental features and allows BLAST searches, but it only hosts a limited amount of sequence data, concerning few reference species. The most similar, and most recent, implementation of a querable system exploiting the Tara Ocean data is the Ocean Gene Atlas [43, 44], which allows users to compare their own sequence data with either the Tara Ocean Microbiome Reference Gene Catalog (for prokaryotes) or the Marine Atlas of Tara Ocean Unigenes (for eukaryotes). This service allows the navigation and visualization of user-defined sets of nucleotide or amino acid sequences that can be explored based on their functional annotation. GLOSSary, in contrast, allows taxonomic based analyses on 16S sequences, supporting investigations on phylogenetic diversity based on this marker. To support the users, the BLAST server embedded in the GLOSSary platform also allows joint analyses versus assembled and unassembled Tara Ocean 16S sequences, and those included in the SILVA database, thus supporting comparative analyses of the different outputs in one shot. This is a straightforward approach to detect novel tags from the Tara Oceans collection which are not included in the SILVA collection.

The GLOSSary platform tackles relevant issues in meta “-omics” from environmental data starting from the organization of heterogeneous 16S rDNA data and from their associated metadata, favouring efficient queries on large amount of information and their analysis by suitable graphical approaches. Instead of replicating already-existing frameworks, GLOSSary also allow for BLAST-like sequence search and comparison, which also integrates a well-established reference database, as well as metadata-informed query of prokaryotic taxa on a global scale.

Although this initial effort is now presented as a framework which embeds the Tara Oceans data, its underlying objective is to expand with additional dataset from similar resources, aiming at a comprehensive collection that could support the exploration of prokaryotic taxonomic diversity integrated with their environmental characterization.

## Conclusions

The current release of the GLOSSary platform aims at favouring the exploration of 16S rRNA gene sequences from large-scale environmental samplings. The database is designed for friendly scalability and multifaceted data uploading. The more conventional sequence based investigations are supported by views that favour environmental metadata exploitation.

The current release of the platform includes 16S data processed from the miTAG collection released by the Tara Ocean expedition and allows not only to identify the location of specific prokaryotic families, but also to investigate their coexistence with user-driven queries, allowing explorations of ecological and phylogenetic issues.

Future efforts will be focused on the inclusion of more 16S data from similar ongoing efforts, to establish a reference resource for the investigation of prokaryotic diversity embedded in the environmental context.

## Availability and requirements

Project name: GLOSSary - the GLobal Ocean 16S Subunit web accessible resource.

Project home page: http://bioinfo.szn.it/glossary/

Operating system(s): Platform independent (web-server).

Programming language: Python (database query system), HTML5 and Javascript (frontend).

Other requirements: none (web-server).

License: none applicable.

Any restrictions to use by non-academics: no restrictions.

## Additional files

Additional file 1: Table summarizing the resulting contigs obtained from the Tara miTAGs assembly. (DOCX 33 kb)

Additional file 2: Visualization of the unevenness of the Tara Ocean sampling effort. (JPG 147 kb)

## Abbreviations

DBMS: Database management system
GLOSSary: The GLobal Ocean 16S Subunit web accessible resource
