# Supplementary material for: GLOSSary: the GLobal Ocean 16S subunit web accessible resource
Source: BMC Bioinformatics. 2018 Nov 30;19(Suppl 15):443. doi: 10.1186/s12859-018-2423-8 (PMC6266928; doi:10.1186/s12859-018-2423-8)
Supplement: Supplementary file 1 — Table summarizing the resulting contigs obtained from the Tara miTAGs assembly. (DOCX 33 kb) [file 12859_2018_2423_MOESM1_ESM.docx]

**Additional File 1. S**ummary of the resulting contigs obtained from the Tara miTAGs assembly.

| **Library name** | **Long contigs (> 900 bp)** | **Long contigs chimera-free** | **Medium contigs (800-900 bp)** | **Medium contigs chimera-free** | **Short contigs** | **Tot contigs** | **Tot miTAGs in library** | **% of mapped miTAGs (97%)** | **Unmapped miTAGs (97%)** |
| --- | --- | --- | --- | --- | --- | --- | --- | --- | --- |
| TARA-004-DCM | 16 | 15 | 6 | 6 | 862 | 884 | 143169 | 21.23 | 112774 |
| TARA-004-SRF | 17 | 14 | 4 | 3 | 574 | 595 | 91124 | 20.24 | 72685 |
| TARA-007-DCM | 18 | 18 | 7 | 7 | 565 | 590 | 77642 | 18.57 | 63221 |
| TARA-007-SRF | 16 | 14 | 3 | 2 | 409 | 428 | 75826 | 21.05 | 59868 |
| TARA-009-DCM | 25 | 22 | 7 | 5 | 795 | 827 | 96015 | 25.81 | 71237 |
| TARA-009-SRF | 27 | 26 | 6 | 6 | 644 | 677 | 147018 | 25.21 | 109948 |
| TARA-018-DCM | 29 | 22 | 6 | 5 | 750 | 785 | 115928 | 26.08 | 85697 |
| TARA-018-SRF | 26 | 21 | 5 | 5 | 826 | 857 | 133643 | 24.53 | 100857 |
| TARA-023-DCM | 29 | 26 | 3 | 3 | 506 | 538 | 53084 | 20.65 | 42122 |
| TARA-023-SRF | 21 | 20 | 6 | 3 | 632 | 659 | 81189 | 23.29 | 62283 |
| TARA-025-DCM | 27 | 23 | 7 | 6 | 762 | 796 | 96926 | 24.33 | 73341 |
| TARA-025-SRF | 28 | 25 | 3 | 3 | 606 | 637 | 133238 | 20.29 | 106206 |
| TARA-030-DCM | 25 | 23 | 5 | 5 | 634 | 664 | 157853 | 21.52 | 123885 |
| TARA-030-SRF | 23 | 20 | 5 | 3 | 463 | 491 | 95690 | 21.35 | 75263 |
| TARA-031-SRF | 22 | 22 | 5 | 5 | 649 | 676 | 124569 | 23.64 | 95125 |
| TARA-032-DCM | 36 | 30 | 3 | 2 | 883 | 922 | 127264 | 30.95 | 87879 |
| TARA-032-SRF | 21 | 18 | 5 | 3 | 519 | 545 | 86601 | 24.02 | 65801 |
| TARA-033-SRF | 24 | 22 | 9 | 8 | 422 | 455 | 69314 | 23.50 | 53024 |
| TARA-034-DCM | 36 | 35 | 9 | 6 | 791 | 836 | 57414 | 28.27 | 41181 |
| TARA-034-SRF | 24 | 22 | 4 | 3 | 412 | 440 | 64924 | 27.07 | 47346 |
| TARA-036-DCM | 15 | 14 | 9 | 6 | 516 | 540 | 100170 | 25.45 | 74677 |
| TARA-036-SRF | 17 | 15 | 2 | 2 | 368 | 387 | 62530 | 25.40 | 46645 |
| TARA-037-MES | 50 | 47 | 13 | 12 | 975 | 1038 | 103619 | 30.64 | 71874 |
| TARA-038-DCM | 25 | 17 | 7 | 6 | 492 | 524 | 100385 | 24.96 | 75325 |
| TARA-038-MES | 25 | 22 | 10 | 8 | 461 | 496 | 54844 | 24.82 | 41232 |
| TARA-038-SRF | 21 | 16 | 4 | 4 | 412 | 437 | 68985 | 26.12 | 50968 |
| TARA-039-DCM | 31 | 25 | 5 | 4 | 419 | 455 | 71132 | 22.15 | 55373 |
| TARA-039-MES | 49 | 45 | 12 | 10 | 619 | 680 | 78278 | 35.63 | 50390 |
| TARA-041-DCM | 15 | 13 | 2 | 2 | 623 | 640 | 76328 | 26.26 | 56285 |
| TARA-041-SRF | 21 | 19 | 7 | 6 | 546 | 574 | 101365 | 22.90 | 78152 |
| TARA-042-DCM | 21 | 19 | 3 | 3 | 458 | 482 | 56690 | 28.14 | 40737 |
| TARA-042-SRF | 16 | 13 | 8 | 6 | 505 | 529 | 97737 | 25.83 | 72493 |
| TARA-045-SRF | 24 | 20 | 8 | 8 | 531 | 563 | 124540 | 28.52 | 89017 |
| TARA-048-SRF | 29 | 22 | 4 | 4 | 459 | 492 | 139279 | 27.62 | 100813 |
| TARA-052-DCM | 17 | 14 | 4 | 3 | 698 | 719 | 96919 | 18.39 | 79095 |
| TARA-052-SRF | 27 | 24 | 4 | 3 | 477 | 508 | 108588 | 23.70 | 82854 |
| TARA-056-MES | 24 | 22 | 4 | 3 | 908 | 936 | 95667 | 21.31 | 75279 |
| TARA-056-SRF | 25 | 22 | 3 | 1 | 591 | 619 | 101902 | 26.67 | 74721 |
| TARA-057-SRF | 31 | 24 | 7 | 6 | 542 | 580 | 100892 | 26.97 | 73681 |
| TARA-058-DCM | 32 | 29 | 5 | 4 | 632 | 669 | 95349 | 23.22 | 73207 |
| TARA-062-SRF | 31 | 27 | 2 | 2 | 531 | 564 | 69585 | 23.93 | 52934 |
| TARA-064-DCM | 28 | 24 | 9 | 9 | 870 | 907 | 161337 | 22.87 | 124432 |
| TARA-064-MES | 15 | 11 | 7 | 6 | 737 | 759 | 63894 | 20.78 | 50617 |
| TARA-064-SRF | 28 | 24 | 6 | 6 | 806 | 840 | 186898 | 26.84 | 136735 |
| TARA-065-DCM | 26 | 21 | 7 | 3 | 727 | 760 | 155299 | 28.80 | 110579 |
| TARA-065-MES | 25 | 21 | 12 | 11 | 1067 | 1104 | 118035 | 20.13 | 94273 |
| TARA-065-SRF | 24 | 18 | 7 | 6 | 472 | 503 | 61426 | 28.09 | 44170 |
| TARA-066-DCM | 16 | 13 | 3 | 2 | 493 | 512 | 39410 | 25.90 | 29204 |
| TARA-066-SRF | 23 | 22 | 3 | 2 | 613 | 639 | 70388 | 26.01 | 52077 |
| TARA-067-SRF | 24 | 19 | 7 | 5 | 514 | 545 | 40590 | 35.61 | 26135 |
| TARA-068-DCM | 20 | 20 | 6 | 6 | 509 | 535 | 56160 | 27.66 | 40625 |
| TARA-068-MES | 35 | 29 | 8 | 7 | 881 | 924 | 115422 | 20.66 | 91571 |
| TARA-068-SRF | 19 | 16 | 7 | 6 | 591 | 617 | 68154 | 25.47 | 50793 |
| TARA-070-MES | 20 | 15 | 6 | 3 | 604 | 630 | 114132 | 16.12 | 95738 |
| TARA-070-SRF | 24 | 22 | 6 | 6 | 429 | 459 | 40905 | 33.11 | 27360 |
| TARA-072-DCM | 23 | 19 | 4 | 4 | 642 | 669 | 49909 | 26.30 | 36785 |
| TARA-072-MES | 18 | 17 | 11 | 8 | 759 | 788 | 73884 | 24.07 | 56103 |
| TARA-072-SRF | 24 | 21 | 7 | 7 | 653 | 684 | 78627 | 23.98 | 59775 |
| TARA-076-DCM | 36 | 31 | 8 | 8 | 626 | 670 | 68102 | 31.03 | 46971 |
| TARA-076-MES | 22 | 18 | 11 | 10 | 1065 | 1098 | 115294 | 22.58 | 89264 |
| TARA-076-SRF | 13 | 11 | 5 | 4 | 475 | 493 | 78051 | 24.33 | 59062 |
| TARA-078-DCM | 36 | 28 | 7 | 7 | 768 | 811 | 87909 | 28.24 | 63085 |
| TARA-078-MES | 19 | 13 | 8 | 8 | 953 | 980 | 83820 | 22.23 | 65191 |
| TARA-078-SRF | 16 | 12 | 0 | 0 | 478 | 494 | 78486 | 20.14 | 62678 |
| TARA-082-DCM | 17 | 15 | 12 | 9 | 533 | 562 | 161515 | 22.78 | 124725 |
| TARA-082-SRF | 13 | 12 | 6 | 5 | 442 | 461 | 72336 | 26.20 | 53385 |
| TARA-084-SRF | 26 | 26 | 6 | 5 | 340 | 372 | 134591 | 21.48 | 105683 |
| TARA-085-DCM | 20 | 16 | 2 | 2 | 256 | 278 | 128279 | 19.19 | 103666 |
| TARA-085-MES | 36 | 29 | 12 | 11 | 787 | 835 | 91144 | 27.82 | 65792 |
| TARA-085-SRF | 17 | 15 | 2 | 1 | 217 | 236 | 163215 | 18.13 | 133631 |
| TARA-093-DCM | 16 | 16 | 3 | 1 | 731 | 750 | 186478 | 43.88 | 104654 |
| TARA-093-SRF | 12 | 11 | 1 | 1 | 469 | 482 | 89154 | 22.46 | 69128 |
| TARA-094-SRF | 24 | 19 | 4 | 3 | 519 | 547 | 127765 | 21.38 | 100451 |
| TARA-096-SRF | 26 | 22 | 4 | 3 | 540 | 570 | 136798 | 19.14 | 110615 |
| TARA-098-DCM | 29 | 25 | 4 | 3 | 634 | 667 | 73002 | 25.23 | 54585 |
| TARA-098-MES | 24 | 19 | 11 | 11 | 1176 | 1211 | 100451 | 20.50 | 79858 |
| TARA-098-SRF | 17 | 17 | 3 | 3 | 429 | 449 | 72312 | 19.72 | 58052 |
| TARA-099-SRF | 11 | 11 | 3 | 2 | 576 | 590 | 96066 | 20.23 | 76632 |
| TARA-100-DCM | 22 | 19 | 6 | 5 | 847 | 875 | 103040 | 23.93 | 78385 |
| TARA-100-MES | 28 | 27 | 8 | 6 | 792 | 828 | 111240 | 26.31 | 81968 |
| TARA-100-SRF | 25 | 24 | 4 | 4 | 504 | 533 | 122482 | 23.56 | 93622 |
| TARA-102-DCM | 30 | 28 | 7 | 6 | 726 | 763 | 112336 | 22.39 | 87188 |
| TARA-102-MES | 28 | 25 | 4 | 4 | 502 | 534 | 56449 | 20.31 | 44986 |
| TARA-102-SRF | 23 | 22 | 5 | 4 | 487 | 515 | 74287 | 24.74 | 55910 |
| TARA-109-DCM | 28 | 24 | 3 | 2 | 512 | 543 | 77265 | 22.53 | 59859 |
| TARA-109-MES | 14 | 11 | 7 | 7 | 891 | 912 | 110154 | 21.43 | 86545 |
| TARA-109-SRF | 27 | 21 | 5 | 5 | 607 | 639 | 128823 | 18.90 | 104471 |
| TARA-110-DCM | 25 | 20 | 7 | 7 | 883 | 915 | 128677 | 22.30 | 99986 |
| TARA-110-MES | 25 | 18 | 9 | 9 | 1120 | 1154 | 114271 | 23.93 | 86925 |
| TARA-110-SRF | 19 | 15 | 6 | 6 | 620 | 645 | 106731 | 23.71 | 81429 |
| TARA-111-DCM | 26 | 22 | 10 | 10 | 845 | 881 | 112059 | 22.53 | 86815 |
| TARA-111-MES | 25 | 22 | 13 | 11 | 891 | 929 | 117215 | 22.39 | 90974 |
| TARA-111-SRF | 17 | 14 | 6 | 6 | 629 | 652 | 115161 | 27.43 | 83575 |
| TARA-112-DCM | 28 | 25 | 11 | 10 | 917 | 956 | 106315 | 23.75 | 81069 |
| TARA-112-MES | 29 | 22 | 10 | 9 | 1047 | 1086 | 82500 | 24.54 | 62254 |
| TARA-112-SRF | 21 | 20 | 11 | 9 | 827 | 859 | 119900 | 19.65 | 96340 |
| TARA-122-DCM | 34 | 29 | 11 | 9 | 933 | 978 | 93757 | 25.65 | 69705 |
| TARA-122-MES | 29 | 25 | 9 | 5 | 1350 | 1388 | 114755 | 20.41 | 91331 |
| TARA-122-SRF | 32 | 25 | 4 | 4 | 557 | 593 | 108073 | 27.59 | 78260 |
| TARA-123-MIX | 50 | 44 | 9 | 8 | 1349 | 1408 | 125791 | 29.42 | 88778 |
| TARA-123-SRF | 41 | 34 | 6 | 6 | 698 | 745 | 86324 | 36.78 | 54571 |
| TARA-124-MIX | 43 | 38 | 6 | 5 | 1155 | 1204 | 141432 | 31.61 | 96730 |
| TARA-124-SRF | 37 | 31 | 7 | 6 | 708 | 752 | 144234 | 25.79 | 107030 |
| TARA-125-MIX | 48 | 41 | 15 | 12 | 1053 | 1116 | 79206 | 28.68 | 56490 |
| TARA-125-SRF | 29 | 23 | 4 | 4 | 620 | 653 | 129135 | 26.13 | 95392 |
| TARA-128-DCM | 36 | 30 | 11 | 4 | 618 | 665 | 76317 | 29.73 | 53627 |
| TARA-128-SRF | 35 | 25 | 11 | 9 | 582 | 628 | 102780 | 31.60 | 70306 |
| TARA-132-DCM | 25 | 21 | 5 | 3 | 1018 | 1048 | 104381 | 27.05 | 76143 |
| TARA-132-MES | 25 | 22 | 17 | 13 | 1054 | 1096 | 112779 | 22.19 | 87755 |
| TARA-132-SRF | 29 | 27 | 3 | 3 | 619 | 651 | 106220 | 21.72 | 83148 |
| TARA-133-DCM | 43 | 36 | 7 | 3 | 665 | 715 | 92255 | 26.87 | 67465 |
| TARA-133-MES | 31 | 25 | 10 | 9 | 1011 | 1052 | 123509 | 23.09 | 94988 |
| TARA-133-SRF | 29 | 24 | 2 | 2 | 744 | 775 | 149404 | 22.98 | 115077 |
| TARA-137-DCM | 43 | 39 | 16 | 14 | 802 | 861 | 118179 | 28.74 | 84217 |
| TARA-137-MES | 51 | 47 | 16 | 15 | 964 | 1031 | 124039 | 33.21 | 82851 |
| TARA-137-SRF | 25 | 23 | 12 | 9 | 566 | 603 | 119711 | 24.54 | 90328 |
| TARA-138-DCM | 22 | 16 | 4 | 4 | 713 | 739 | 68607 | 23.40 | 52550 |
| TARA-138-MES | 45 | 42 | 12 | 12 | 1041 | 1098 | 100842 | 28.19 | 72419 |
| TARA-138-SRF | 34 | 27 | 9 | 8 | 646 | 689 | 111087 | 21.77 | 86900 |
| TARA-140-SRF | 30 | 23 | 7 | 6 | 733 | 770 | 115773 | 24.56 | 87336 |
| TARA-141-SRF | 32 | 28 | 8 | 8 | 574 | 614 | 77211 | 24.68 | 58153 |
| TARA-142-DCM | 37 | 32 | 4 | 4 | 818 | 859 | 100452 | 27.45 | 72878 |
| TARA-142-MES | 27 | 21 | 10 | 4 | 942 | 979 | 111238 | 19.03 | 90065 |
| TARA-142-SRF | 44 | 35 | 6 | 5 | 794 | 844 | 109445 | 26.64 | 80288 |
| TARA-145-MES | 26 | 21 | 11 | 10 | 1095 | 1132 | 98337 | 23.34 | 75382 |
| TARA-145-SRF | 28 | 23 | 8 | 7 | 922 | 958 | 84116 | 29.15 | 59597 |
| TARA-146-MES | 32 | 27 | 10 | 9 | 979 | 1021 | 83957 | 24.10 | 63726 |
| TARA-146-SRF | 33 | 30 | 7 | 7 | 970 | 1010 | 118221 | 27.82 | 85331 |
| TARA-148-SRF | 44 | 38 | 13 | 11 | 863 | 920 | 95080 | 27.50 | 68932 |
| TARA-148b-MES | 31 | 25 | 11 | 9 | 1398 | 1440 | 118053 | 27.67 | 85389 |
| TARA-149-MES | 21 | 20 | 15 | 15 | 1123 | 1159 | 109336 | 20.91 | 86470 |
| TARA-149-SRF | 26 | 22 | 10 | 9 | 882 | 918 | 120367 | 29.83 | 84457 |
| TARA-150-DCM | 27 | 25 | 4 | 4 | 863 | 894 | 118668 | 21.94 | 92629 |
| TARA-150-SRF | 19 | 18 | 6 | 6 | 839 | 864 | 123658 | 24.54 | 93316 |
| TARA-151-DCM | 26 | 22 | 8 | 8 | 1081 | 1115 | 104170 | 25.84 | 77256 |
| TARA-151-SRF | 21 | 16 | 9 | 7 | 773 | 803 | 122930 | 21.68 | 96281 |
| TARA-152-MES | 35 | 28 | 17 | 15 | 1151 | 1203 | 109195 | 20.70 | 86587 |
| TARA-152-MIX | 22 | 18 | 4 | 2 | 828 | 854 | 113269 | 23.42 | 86742 |
| TARA-152-SRF | 28 | 27 | 7 | 5 | 690 | 725 | 92320 | 22.50 | 71544 |
