# Supplementary figures and images for: GLOSSary: the GLobal Ocean 16S subunit web accessible resource
Source: BMC Bioinformatics. 2018 Nov 30;19(Suppl 15):443. doi: 10.1186/s12859-018-2423-8 (PMC6266928; doi:10.1186/s12859-018-2423-8)

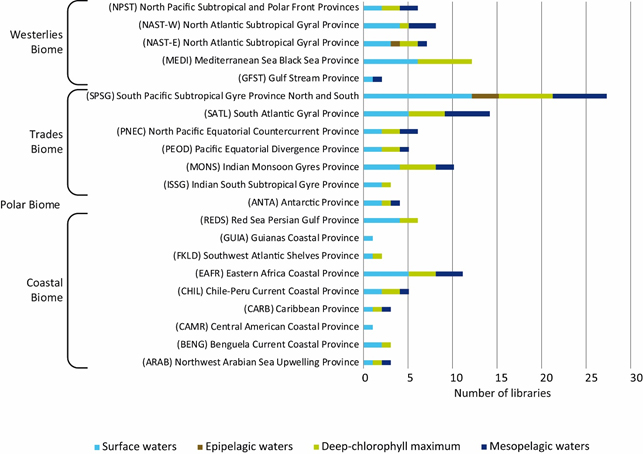

Supplement: Supplementary file 2 — Visualization of the unevenness of the Tara Ocean sampling effort. (JPG 147 kb) [file 12859_2018_2423_MOESM2_ESM.jpg]
